# Abdominal Dropsy

**Published:** 1856-03

**Authors:** 


### SELECTIONS.

*Hospital Cases.*

Pennsylvania Hospital, *Jan.*, 1856.

1. Abdominal Dropsy.—A report is made of the following case, not because of any unusual interest during its progress, but on account of an unusual pathological condition, exposed on post-mortem examination.

*Dec.* 8. Man, 35. Exsanguine appearance of a victim of miasmatic disease—œdematous extremities—abdomen swollen to the dimensions of a female in the eighth month of pregnancy —superficial veins turgid—spleen much enlarged, encroaching on the lungs, and of scirrhoid hardness. Liquid is effused into right pleura, and the pericardium is burdened with it. The urine presents no evidences of albuminuria; alvine dejections normal. Patient complains of abdominal pain and dyspnœa. The inferential diagnosis is, that scirrhus of the spleen, and a sub-inflammation of the peritoneal cavity exist. The case is one hardly likely to recover. The treatment to pursue is that which will relieve the most apparent symptom—dropsical effusion.

*Jan.* 5. The patient has been placed on the usual diuretic remedies, with reference only to the amelioration of his symptoms—an incurable organio affection complicates the case. Cream of tartar and juniper-berry tea, alternately with elaterium and digitalis, have been employed with some advantage, at times, but there is no permanent gain. A very dry tongue indicated a condition of the alimentary canal requiring the use of oil of turpentine.

*Jan.* 12. *Autopsy.*—The *fons et origo* of the mischief is the liver and not the spleen; while the latter appears as large as an average sized liver, the former is reduced to *one-fourth* its normal dimensions, and is cirrhosed; in form it is remarkably altered—presenting no thin anterior border, but contracted into a knotty mass. Little granulated masses jut out, over the whole surface of the organ, giving it the puckered appearance of "hobnail liver," of which it is an excellent example. The cut surface is of a yellowish hue, dotted with innumerable red points; blood flows from the larger vessels, but it has not been able to penetrate into the small ramifications. The nature of this affection is not fully determined. Laennec bestowed on it the name *Cirrhosis*, and supposed a peculiar product, analogous to that of tubercle in the lung, to be deposited here. This opinion is now abandoned. Beneath the microscope, there is found to be an hypertrophy and contraction of the capsule of Glisson, surrounding each acinus, and giving sheaths to all the vessels. The acini remain unaffected in their secreting functions, unless their supply of blood is cut off by the obliteration of vessels; hence we find that jaundice was not occasioned, because a limited portion of this liver was still actively discharging its duty. The same fibroid degeneration is observed in the kidney and pancreas, and in nature is not inflammatory, nor the result of that process.

This condition of the liver will explain all the other pathological evidences. Gall-bladder filled with bile. Peritoneum and mucous membrane of intestine injected with blood. Pancreas of scirrhous hardness, cutting like cartilage. Lungs too dark. Heart atrophied, much diminished in its cavity. Serous sacs filled with liquid.

*Jan.* 9. MENINGITIS.—Young man, brought in day before yesterday; had been taken with convulsions, which were preceded by a two days' sickness.

Since the convulsions, has lain in a state of coma, with occasional spasmodic movements. Answers no questions; pupils contracted, and eyes red. Will not protrude the tongue; patient is sensible to inconvenience, as is proved by partial voluntary muscular action, when surface is exposed to the cold; this is dependent on no cerebral operation of the will, but the result of reflex action, the same as is noticed in decapitated animals, the alligator, &c.

Pulse very feeble; there is no positive depression, and though a meningeal inflammation exists, inducing irritation of the nerve centres, it is too late to use active antiphlogistic measures. Should have been bled largely in the commencement of the attack. Now we can have little expectation of recovery. Apply blisters repeatedly to scalp. Blisters to feet. Calomel, grs. x. Turpentine enemata, frequently repeated during the day.

*Jan.* 12. Improvement since the above treatment. Breathes with tolerable regularity. Face has not so dinged a hue; pupils still contracted; dryish condition of skin; pulse feeble, but not small; has articulated a few words, and is more sensible this morning than at any previous time; indisposed to move left arm, which is an unfavorable sign, indicating a complication of brain substance.

Administer a supporting diet; if convulsions or coma increase, apply a few leeches about the temples; keep bowels open with turpentine enemata; pil. hydrarg. gr. ij four times a day; continue the irritation of scalp.

*Jan.* 19. *Autopsy. Brain*—Great congestion of pia mater, redness beneath arachnoid, which is opaque, with effusion of coagulable lymph. Paralysis of left arm was due to change in the substance of brain itself. Small blood-clots in various positions in the right lobe, and one spot is decidedly soft, creamy and disorganized. In left hemisphere no apparent change. Acute cerebritis and meningitis coexisted.

PHILADELPHIA HOSPITAL, *January*, 1856.

This institution has, during the past winter, been opened for clinical instructions, and lectures have been delivered twice a week. We shall occasionally present reports of these lectures.

